# Synthesis of Biotinylated PAMAM G3 Dendrimers Substituted with *R*-Glycidol and Celecoxib/Simvastatin as Repurposed Drugs and Evaluation of Their Increased Additive Cytotoxicity for Cancer Cell Lines

**DOI:** 10.3390/cancers14030714

**Published:** 2022-01-29

**Authors:** Konrad Wróbel, Stanisław Wołowiec, Joanna Markowicz, Elżbieta Wałajtys-Rode, Łukasz Uram

**Affiliations:** 1 Medical College, Rzeszów University, 1a Warzywna Str., 35-310 Rzeszów, Poland; konradwrobel300@gmail.com (K.W.); swolowiec@ur.edu.pl (S.W.); 2 Faculty of Chemistry, Department of Inorganic and Analytical Chemistry, Rzeszów University of Technology, 6 Powstańcow Warszawy Ave., 35-959 Rzeszów, Poland; jmarkowicz@stud.prz.edu.pl; 3 Faculty of Chemistry, Warsaw University of Technology, 75 Koszykowa Str., 00-664 Warsaw, Poland; ewalajtys@ch.pw.edu.pl

**Keywords:** drug delivery system, biotinylated, *R*-glycidylated PAMAM G3 dendrimers, celecoxib, simvastatin, molecular size, cytotoxicity, human squamous carcinoma (SCC-15-15), human glioblastoma (U-118 MG), normal human fibroblasts (BJ)

## Abstract

**Simple Summary:**

New anti-cancer drugs can be created through the combination of well-known substances acting on different molecular targets. Repurposed drugs selected in this way can then be attached to special carriers that have specific, high affinity for cancer cells. In this study, two anti-glioma drugs, celecoxib and simvastatin, were chosen and linked to the PAMAM G3 dendrimer targeted to cancer cells by attached biotin and *R*-glycidol. In vitro studies performed with human glioblastoma (U-118 MG) and squamous cell carcinoma (SCC-15) cells revealed that dendrimer conjugate containing both celecoxib and simvastatin was 20–50 times more potent than either drug administered alone or in combination. Thus, the obtained combined conjugate can be considered as a potential candidate for a new therapy of malignant glioblastoma.

**Abstract:**

Recent achievement in anticancer therapy considers the application of repurposed drugs in optimal combinations with the use of specific carriers for their targeted delivery. As a result, new optimized medications with reduced side effects can be obtained. In this study, two known anticancer drugs, celecoxib and/or simvastatin, were conjugated covalently with PAMAM G3 dendrimer and tested in vitro against human squamous carcinoma (SCC-15-15) and glioblastoma (U-118 MG) cells, as well as normal human fibroblasts (BJ). The obtained conjugates were also substituted with biotin and *R*-glycidol to increase their affinity for cancer cells and were characterized with NMR spectroscopy and dynamic light scattering technique. Conjugates furnished with two celecoxib and four simvastatin residues revealed the very high effectiveness and dramatically decreased the SCC-15 and U-118 MG cell viability at very low concentrations with IC_50_ equal to about 3 µM. Its action was 20–50-fold stronger than that of either drug alone or as a mixture. Combined conjugate revealed also additive action since it was 2–8-fold more effective than conjugates with either single drug. The combined conjugate revealed rather low specificity since it was also highly cytotoxic for BJ cells. Despite this, it may be concluded that biotinylated and *R*-glycidylated PAMAM G3 dendrimers substituted with both celecoxib and simvastatin can be considered as a new perspective anticancer agent, effective in therapy of malignant, incurable glioblastomas.

## 1. Introduction

**Scheme 1:**
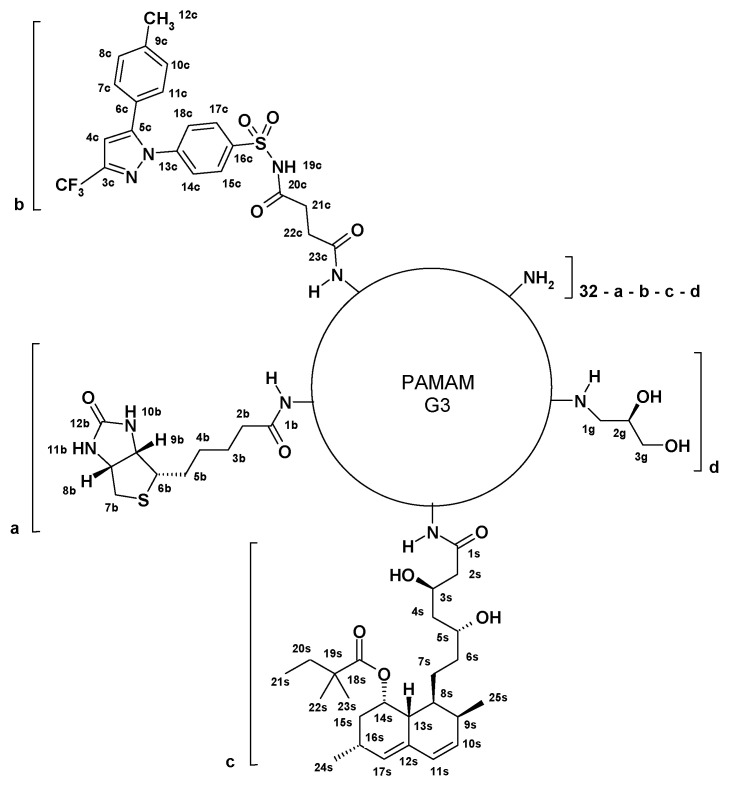
Schematic formula of PAMAM G3 (G3) conjugates with biotin (B), celecoxib (C), simvastatin (S), and *R*-glycidol (gl). The following conjugates were tested: G3^2B6C8gl^ (a = 2; b = 6, c = 0, d = 8), G3^2B4S14gl^ (a = 2; b = 0, c = 4, d = 14), G3^2B2C4S12gl^ (a = 2; b = 2, c = 4, d = 12), and G3^2B14gl^ (a = 2; b = 0, c = 0, d = 14).

The new approach in the search for effective cancer therapy is the use of repurposed drugs in various combinations together with their targeted delivery for optimization of therapeutic effects with concomitant decrease of side effects. Well-characterized carriers of drugs targeted into cancer, the biotinylated PAMAM G3 dendrimers, were chosen. Their ability for introduction of cargo to cancer cells has been well documented [1]. Among many possible candidates used in clinics in cancer treatment, we selected two well-recognized and characterized drugs, namely, celecoxib and simvastatin. There is a plethora of publications concerning the antineoplastic effects of both substances and their molecular mechanisms of action. Celecoxib (C) as an inhibitor of cyclooxygenase-2 (COX-2) exhibits anti-inflammatory properties reducing the promotion of the cancer-induced microenvironment [2]. The anticancer effect of C attached to the biotinylated PAMAM G3 dendrimers was documented for U-118 MG glioma and squamous carcinoma (SCC-15) cancer cell lines [3,4,5,6,7]. Statins are also well characterized as anticancer agents. Statins upregulate or inhibit diverse signaling pathways, resulting in the cytotoxicity and apoptosis of cancer cells. Affected signaling pathways lead to differentiation of cancer cells; induction of oxidative stress; cell cycle arrest; autophagy; and suppression of cancer stemness, proliferation, metastasis, and angiogenesis [8]. Statins inhibit the 3-hydroxy-3-methyl-glutaryl-CoA (HMG-CoA) reductase that is the rate-limiting enzyme in the cholesterol biosynthesis pathway [9]. They reduce mevalonate synthesis, and, consequently, farnesylation and geranylgeranylation processes are suppressed. As an elevated activity of mevalonate (MVA)/cholesterol biosynthetic pathway is linked to the initiation and progression of many types of cancers, statins have been extensively used in cancer therapy [10]. One of the often applied statins is the lipophilic substance simvastatin (S) [11]. The antitumor effects of S involves overlapping molecular pathways and different subcellular structures. Beyond the inhibitory effect on cholesterol production, S has the pleiotropic effects including anti-proliferation, anti-apoptosis, anti-angiogenesis, and prevention of metastasis due to the activation of certain proteins that interfere with important signaling pathways. It has been documented that S significantly suppresses the expression of the tumor-promoting cytokines and mediators, including the transforming growth factor (TGF)-β1, vascular endothelial growth factor (VEGF), and interleukins (IL-8 and IL-6), inhibiting ovarian cancer cell growth [12]. In prostate cancer cells, S has been shown to enhance the intrinsic apoptosis by activation of pro-apoptotic and inhibition of anti-apoptotic proteins and simultaneously to stimulate extrinsic apoptosis by increasing of the TNF-alpha, Fas-L, Traf, and caspase 8 [8]. It has to be noticed that during statin monotherapy, patients suffer from many side effects, including liver toxicity, myopathy, proteinuria, or hematuria and increased diabetes risk [9]. However, many investigations in vitro and in vivo documented effects of S against various diseases including cancer [11] and revealed that S can enhance the response to conventional anticancer therapies when administered in combination with other drugs or in combination with various nanostructures. This includes encapsulation S in the poly (lactic-co-glycolic acid) microspheres [13] or G4 PAMAM dendrimers with three different functionalities (NH_2_, OH, and PEG) [14,15] (as well as G4 PAMAM-OH dendrimers with glycosylated surface) [16]. Nanoparticles for simvastatin delivery has been recently reviewed [17]. Investigations of S in combination revealed that S and temozolomide used simultaneously significantly increased apoptosis in glioblastoma cells [18]. Studies on the combination of S and C performed by Gehrke et al., 2017 on head and neck squamous cell carcinoma cells (HNSCC) revealed only small additive and no synergistic effect with the combination of 50 µM S and 40 µM C [19]. Thus, it was reasonable to construct the targeted carrier that will introduce both S and C into cancer cells, expecting that it will cause significant decrease of cytotoxic concentrations of both drugs and promote elimination of side effects. Therefore the one-macromolecule-two drugs conjugate, based on PAMAM G3 was constructed (Scheme 1) and tested against cancer and normal cell lines in vitro. Additionally the conjugate was equipped with biotin in order to increase the affinity of a conjugate to cancer cell membrane according to formerly determined strategy [20]. Furthermore, the remaining primary amine groups of PAMAM G3 core were substituted with *R*-glycidol. Recently, we have found that G2 and G3 PAMAM dendrimers covered with *R*-glycidol entered cells faster and showed higher toxicity for cancer than normal cells than *S*-glicidol-covered analogues [21].

## 2. Materials and Methods

### 2.1. Reagents

Simvastatin, ethylenediamine, methyl acrylate, succinic anhydride, α-D-glucoheptono-1,4-lactone (GHL), carbonyl diimidazole (CDI), 2-chloro-1-methylpyridinium iodide, 4-dimethylaminopyridine (DMAP), triethylamine (TEA), *R*-glycidol, dimethyl sulfoxide (DMSO), and other reagents used in syntheses were obtained from Merck KGaA (Darmstadt, Germany). Celecoxib was purchased from Abcam GmbH (Karlsruhe, Germany), while biotin *N*-hydroxysuccinimide ester (NHS-Biotin) was obtained from AmBeed (Arlington Hts, IL, USA). Spectra/Por^®^ 3 RC dialysis membrane (cellulose, MW_cutoff_—3.5 kD) was provided by Carl Roth GmbH and Co. KG (Karlsruhe, Germany).

### 2.2. Biochemical Reagents, Cell Lines and Materials

Human glioblastoma (U-118 MG, ATCC^®^ HTB-15), human squamous cell carcinoma (SCC-15, ATCC^®^ CRL-1623), and human normal fibroblast (BJ, ATCC^®^ CRL-2522) cell lines were purchased from the American Type Culture Collection (ATCC, Manassas, VA, USA). Dulbecco’s modified Eagle’s media (DMEM and DMEM/F-12), Eagle’s minimum essential medium (EMEM), and fetal bovine serum (FBS) were obtained from Corning Inc. (New York, NY, USA). Penicillin and streptomycin solution, phosphate-buffered saline (PBS) with and without magnesium, and calcium ions were provided by Thermo Fisher Scientific Inc. (Waltham, MA, USA). Trypsin-EDTA solution, hydrocortisone, 0.33% neutral red solution (3-amino-N-dimethylamino-2-methyl-phenazine hydrochloride), XTT sodium salt (2,3-bis[2 -methoxy-4-nitro-5-sulfophenyl]-2H-tetrazolium-5-carboxanilide inner salt), and phenazinemethosulfate (PMS) were purchased from Merck KGaA (Darmstadt, Germany). Cell cultures dishes and materials were from Corning Incorporated (Corning, NY, USA), Greiner (Kremsmünster, Austria), or Nunc (Roskilde, Denmark).

### 2.3. Syntheses

PAMAM G3 dendrimer (G3) was obtained according to the original procedure of Tomalia et al. [22]. G3 was converted into biotin attached conjugate, G3^2B^, using two equivalents of NHS-biotin in DMSO at 130 µmoles scale and purified by dialysis according to the protocol described before [23,24].

#### 2.3.1. Preparation of G3^2B6C8gl^ Conjugate with Succinyl-Celecoxib

**Figure 1:**
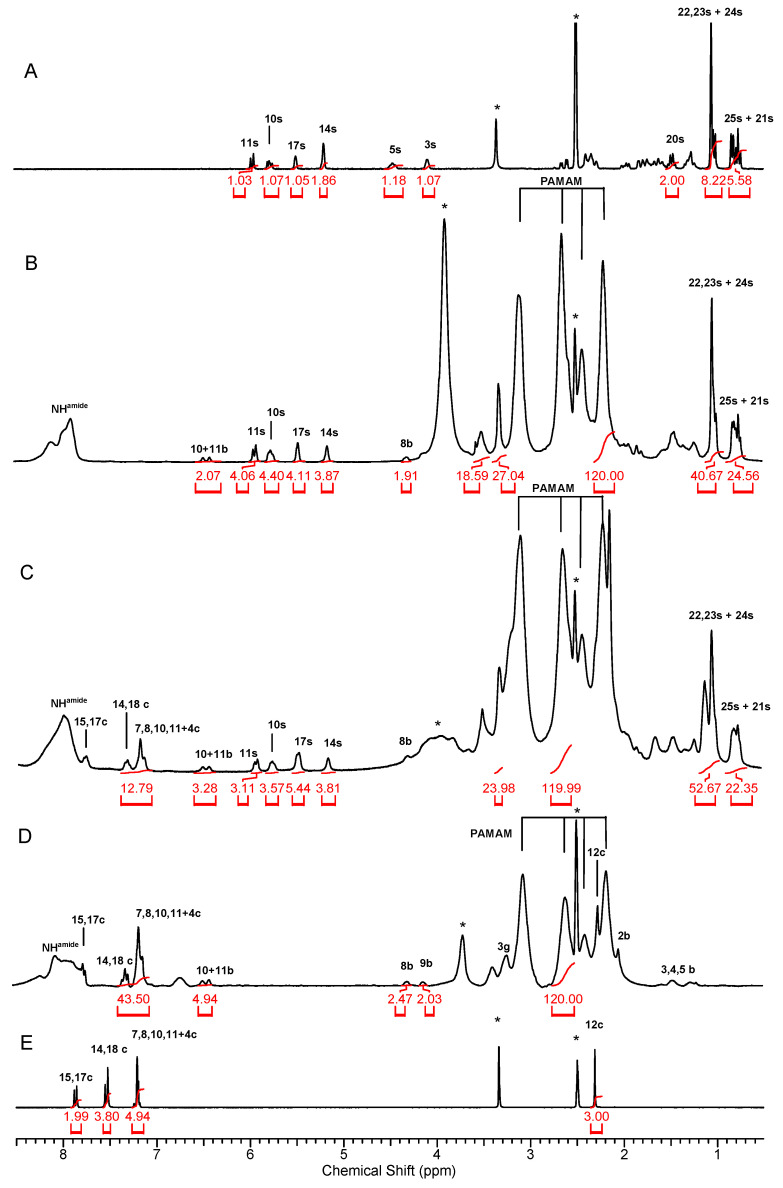
The ^1^H NMR spectra of simvastatin (**A**), G3^2B4S14gl^ (**B**), G3^2B2C4S12gl^ (**C**), G3^2B6C8gl^ (**D**), and celecoxib (**E**) in DMSO-d_6_. Residual HD_2_CS(O)CD_3_ and HDO peaks are labeled with asterisks *. The PAMAM CH_2_ resonances centered at 2.63 ppm or 2.20 were used as internal integration reference (the integral intensity of both resonances corresponds to [120H]).

The G3^2B^ conjugate was substituted with celecoxib according to previously published method [25] with some modifications as follows: celecoxib (318.0 mg, 834 µmoles) was converted into succinate-celecoxib (SC) by reaction with succinic anhydride (93.6 mg, 935 µmoles) in acetonitrile (8 mL) in the presence of 1 mL TEA under reflux for 6 h. Then, solvents were evaporated under reduced pressure, a solid residue was dissolved in dimethylsulfoxide (DMSO, 11 mL), and SC in 2 mL of DMSO (151 µmoles) was activated with Mukaiyama reagent. Thus, solid 4-(dimethylamino)pyrimidine (DMAP, 185 mg, 1.51 mmoles) and then 2-chloro-,N-methyl pyridinium iodide (193 mg, 755 µmoles) were added, and the mixture was stirred vigorously for 4 h at room temperature and then added into G3^2B^ in 5 mL DMSO (158 mg, 21.5 µmoles). The mixture was heated at 50 °C for 12 h, then transferred into cellulose dialytic bag (MW_cutoff_ = 3.5 kDa) and dialyzed for 3 days against water (7 times 5 L). Water was removed on a rotary evaporator under reduced pressure (20 mbar), the solid residue was dried overnight in vacuo (<2 mbar), and 136 mg of product was obtained and characterized by the ^1^H NMR as conjugate of PAMAM G3 with average 2 amide-attached biotin residues and 6 amide-linked SC residues, G3^2B6C^. Overall yield was 13.4 µmoles (62.3%), considering the theoretical MW equal 10,129 Da. Then, G3^2B6C^ (13.4 µmoles) was dissolved in 3 mL DMSO and 5 mL methanol. To this solution, we added *R*-glycidol (40 µL, 44.6 mg, 600 µmoles) stepwise, and the mixture was left at room temperature for 12 h. Afterwards, the mixture was dialyzed against water, and the solid residue was isolated as before. The product was identified as conjugate with additional eight 2(*R*),3-dihydroxypropyl residues attached to 8 amine groups out of 24 available in G3^2B6C^ substrate. Yield of G3^2B6C8gl^: 115 mg, 10.7 µmoles, 80% considering the average MW equal 10,722 Da. The stoichiometry of macromolecular product was identified by the ^1^H NMR spectroscopy (Figure 1).

#### 2.3.2. Conjugation of Simvastatin to G3 PAMAM

Simvastatin (53.5 mg, 128 µmoles) was dissolved in 2 mL methanol. To this solution, we added 38.5 mg of CDI (237 µmoles). The formation of white precipitate was observed, which disappeared within 15 min of stirring at room temperature. Then, the mixture was heated at 42 °C for 3 h. This solution was then added dropwise into a methanolic solution of G3 containing 69.8 mg (10.1 µmoles). The mixture was heated at 42 °C for 12 h. Afterwards, the mixture was transferred into dialytic bag, dialyzed against water for 3 days, and isolated as described above. Water was removed under reduced pressure, and the solid residue was dried in vacuo. Although the product was well soluble in water, special care was taken at the isolation step due to vast foaming of the aqueous solution of the product, which was isolated at the yield 45.6 mg (38% recalculated for average theoretical MW = 11,931 Da). The product was identified by ^1^H NMR spectroscopy as G3^12S^ (for detailed analysis of 1-D ^1^H and 2-D ^1^H-^1^H COSY spectrum, see Figure S1). The ^1^H and ^13^C resonances were assigned on the basis of HSQC and HMBC spectra and chemical shifts of the resonances are collected in Table S1.

#### 2.3.3. Preparation of G3^2B4S14gl^ Conjugate with Simvastatin

Simvastatin (S, 62.9 mg, 150 µmoles) was dissolved in 3 mL methanol. To this solution 100 µL TEA was added, followed by stepwise addition of carbonyl diimidazole (CDI, 48.5 mg, 300 µmoles), while vigorous stirring and allowed for a reaction for 1 h at room temperature. This solution was added dropwise to G3^2B^ (179.7 mg, 24.4 µmoles) in methanol. The mixture became turbulent, and after 30 min stirring, all materials dissolved. The mixture was kept at room temperature for 4 h, then *R*-glycidol (80 µL, 89 mg, 1200 µmoles) was added dropwise; following this, the mixture was stored for 12 h and then dialyzed against water as before. The solid product was isolated and identified as G3^2B4S14gl^ by the ^1^H NMR spectroscopy (Figure 1B). The isolated yield was 107.5 mg, 10.7 µmoles, and 43.8% for theoretical molecular weight equal to 10,001 Da.

#### 2.3.4. Preparation of G3^2B2C4S12gl^ Conjugate with Celecoxib and Simvastatin

The ternary conjugate of G3^2B^ with SC and simvastatin was obtained in the two-step strategy. First, the 75 µmoles SC in 2 mL DMSO was activated with 0.225 mmole of 2-chloro-N-methyl pyridinium iodide (57 mg) in the presence of 0.450 mmole DMAP (55 mg). This solution was added into 220 mg G3^2B^ (30 µmoles) in 2 mL DMSO, and the mixture was heated at 50 °C for 12 h. After dialytic purification and removal of solvents, the isolated solid product (207.1 mg, 25 µmoles, 83.3% yield) was identified as G3^2B2C^ by ^1^H NMR spectroscopy.

Then, 207.1 mg of G3^2B2C^ (25 µmoles) was dissolved in methanol (7 mL), and to this solution, we dropwise added simvastatin (52.3 mg, 125 µmoles), preliminarily activated with 40.5 mg CDI (250 µmoles in 2 mL methanol and 100 µL TEA). The mixture was kept at 60 °C for 4 h. Then, *R*-glycidol (80 µL, 89 mg, 1200 µmoles) was added dropwise, and the mixture was stored for 12 h at ambient temperature and then dialyzed against water as before. Solvents were removed under reduced pressure and the solid residue analyzed by the ^1^H NMR spectroscopy. The average stoichiometry of the product was determined as G3^2B2C4S12gl^ (Figure 1C). Yield: 189 mg, 17.5 µmoles, 70 % calculated for theoretical molecular weight of the product equal to 10,776 Da.

#### 2.3.5. Synthesis of G3^2B14gl^ Conjugate

*R*-glycidol (12.2 mg; 165 µmoles in 0.5 mL methanol) was added dropwise to 75 mg G3^2B^ (10.2 µmoles) in 1 mL methanol with vigorous stirring and left at ambient temperature for 2 days. Then, the mixture was transferred to dialytic tube and dialyzed against water for 3 days. The product was dried and identified by ^1^H NMR spectroscopy as G3^2B14gl^, as described previously [12]. The molecular size and zeta potential for the carrier and conjugates were determined by DLS (Table 1).

### 2.4. Methods

#### 2.4.1. NMR Spectroscopy

The 1-D ^1^H and ^13^C NMR spectra and 2-D ^1^H-^1^H correlations spectroscopy (COSY), ^1^H-^13^C heteronuclear single quantum correlation (HSQC), and heteronuclear multiple bond correlation (HMBC) spectra were recorded in DMSO-d_6_ using a Bruker 300 MHz instrument (Rheinstetten, Germany) at College of Natural Sciences, University of Rzeszów.

#### 2.4.2. Conjugate Size and ζ Potential Measurements

Size and ζ potential of G3^2B14gl^, G3^12S^, G3^2B6C8gl^, G3^2B4S14gl^, and G3^2B2C4S12gl^ conjugates were measured using dynamic light scattering technique at pH 5 (0.05 M acetate buffer) and in water using Zetasizer Nano instrument (Malvern, UK) for 1 mg/mL samples (0.7–1.0 mM solutions). The size of conjugates at pH 7 and 5 and values of zeta potential for conjugates and G3^2B14gl^ are collected in Table 1.

#### 2.4.3. Cell Culture

Human glioblastoma cells U-118 MG (HTB-15, ATCC) were cultured in DMEM, human squamous carcinoma cells SCC-15 (CRL-1623 ATCC) were grown in DMEM/F-12 supplemented with hydrocortisone (400 ng/mL), and normal human skin fibroblasts BJ (CRL-2522 ATCC) were cultured in EMEM. All culture media were supplemented with 10% FBS and 100 U/mL penicillin 100 µg/mL streptomycin solution. All cell lines were incubated at 37 °C in humidified 95% air/5% CO_2_ with media changed every 2–3 days. Cells were passaged at 70–85% confluence after trypsinization with 0.25% trypsin-EDTA in PBS (calcium and magnesium free). Cell morphology was evaluated with Nikon TE2000S Inverted Microscope with phase contrast (Tokyo, Japan). Number and viability of cells were estimated by trypan blue exclusion test using Automatic Cell Counter TC20 (BioRad Laboratories, Hercules, CA, USA) or Neubauer chamber. All assays were performed in triplicate in three independent experiments.

#### 2.4.4. Cytotoxicity Assays

BJ, SCC-15, and U-118 MG cells were seeded in flat-bottom 96-well culture plates in triplicate (100 µL cell suspension per well) at a density of 1 × 10^4^ cells per well and allowed to attach for 24 h. The stock solution of 200 mM celecoxib and simvastatin or 5.9 mM G3^2B6C8gl^, 8.1 mM G3^2B4S14gl^, or 8.9 mM G3^2B2C4S12gl^ in DMSO were used to make working solutions in appropriate media with a range of increasing concentrations: celecoxib and simvastatin from 0 to 200 µM (100 µL/well), PAMAM conjugates with drugs from 0 to 12 µM (100 µL/well). The 9.7 mM stock solution of dendrimer vehicle G3^2B14gl^ in distilled water was filtered with sterile syringe filters (0.22 µm) and used to 0–200 µM working solutions (100 µL/well). All cell lines were incubated with working solutions for 24 h in 37 °C. After that, the neutral red (NR) and XTT assays were performed as described earlier [26].

#### 2.4.5. Statistical Analysis

To estimate the differences between treated and non-treated control samples, we performed statistical analysis using the non-parametric Kruskal–Wallis test due to the lack of a normal distribution of data in the studied groups. *p* < 0.05 was considered statistically significant. Calculations were performed with Statistica 13.3 software (StatSoft, Tulsa, OK, USA).

## 3. Results and Discussion

### 3.1. Syntheses and Characterization of Conjugates

**Scheme 2:**
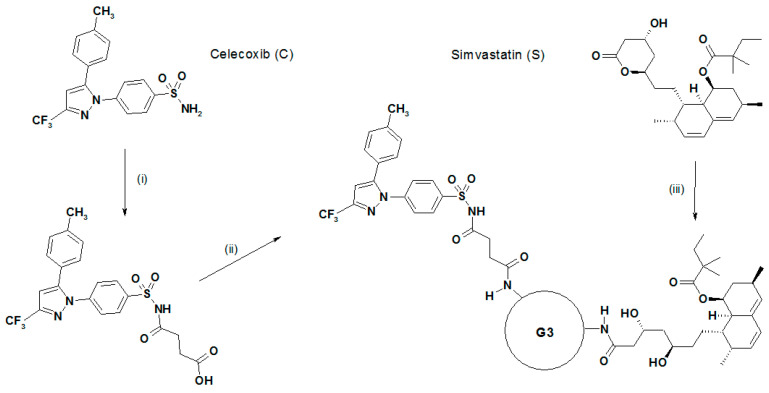
Functionalization of celecoxib and simvastatin: (i) succinic anhydride in acetonitrile and triethylamine, (ii) 2-chloro-N-methylpyridine iodide in the presence of 4-(dimethylamino)pyridine in DMSO, (iii) carbonyl diimidazole in methanol.

Third-generation poly(amidoamine) dendrimer substituted with two equivalents of amide-attached biotin was used as macromolecular substrate (G3^2B^) to obtain celecoxib and simvastatin conjugates (Scheme 1). Celecoxib was converted into succinyl-celecoxib (SC) as it was described in [25] and attached through amide-succinyl linker to G3^2B^ using Mukaiyama reagent [27] to obtain highly substituted (G3^2B6C^) and low-substituted (G3^2B2C^) derivatives as was elaborated previously (Scheme 2) [3,28].

Simvastatin (S) was covalently attached via amide bond to both G3^2B^ and G3^2B2C^ to obtain equally substituted conjugates G3^2B4S^ and G3^2B2C4S^, respectively. This conversion was elaborated from the beginning. Namely, we monitored a reaction between S and ethylenediamine (en) by the ^1^H NMR spectroscopy in deuterated methanol (Figure S1). We found that S lactone ring opening occurred within 4 h of refluxing 20 mM S and 23 mM en in methanol with formation of amide linkage between S and amine group of en. The addition of carbonyl diimidazole, which is a known carboxylate group-activating agent [25], sped up the reaction twice. Therefore, we applied this reagent to test the S addition into PAMAM G3 dendrimer (Scheme 1) in methanol. We obtained highly substituted G3^12S^ derivative and characterized it by the ^1^H NMR and 2-D ^1^H-^1^H COSY spectroscopy in DMSO-d_6_ (Figure S1). The most dramatic changes in the ^1^H NMR spectra were observed for 3s and 5s protons. These resonances shifted upfield from 4.20 ppm and 4.05 ppm in S (for spectral assignment, see Figure S3 and previously performed assignments [29]) into 3.65 ppm and 4.17 ppm in G3^12S^, respectively. All the ^1^H resonances assigned to lactone ring in S (except 5s) shifted upfield in the G3^12S^. Moreover, the ^13^C NMR resonances of lactone form of S shifted considerably in the ^13^C NMR spectrum of G3^12S^. The ^13^C resonances were assigned on the basis of HSQC and HMBC experiments (Table S1).

Using the same reaction conditions, we added four equivalents of S into the G3^2B^ and G3^2B2C^ to obtain G3^2B4S^ and G3^2B2C4S^, which were not isolated from methanol. Further, we blocked the remaining amine groups in G3^2B6C^, G3^2B2C4S^, and G3^2B4S^ by reaction of the conjugates with excess of *R*-glycidol in comparable conditions in methanol. Surprisingly, the remaining free amine groups of the starting conjugates were only partially substituted, as was observed before in the case of G3-fluorescein and G3-rhodamine conjugates [21]. On the basis of the integral intensity of the 3g resonance centered at around 3.32 ppm related to internal reference PAMAM resonance of [120H] intensity, we found that glycidylated conjugates were: G3^2B6C8gl^, G3^2B2C4S12gl^, and G3^2B4S14gl^, as determined by the ^1^H NMR spectra (Figure 1B–D, respectively, Table S1).

The resonances in the ^1^H NMR spectrum of S (Figure 1A) in DMSO-d_6_ were assigned on the basis of COSY experiment (Figure S3). Amide conjugation of S into G3^2B4S14gl^ resulted in a considerable upfield shift of 5s and 3s protons, which are hidden under high-intensity residual HDO resonance (Figure 1B and Figure S4), whereas 10s, 11s, 14s, and 17s remained almost unchanged in the ^1^H NMR spectrum of G3^2B4S14gl^ related to S. However, the 3s and 5s were undoubtedly identified in the spectrum of G3^12S^ (Figure S2). The integral intensities of 10s, 11s, 14s, and 17s resonances corresponded to four S substituents in the G3^2B4S14gl^ conjugate in relation to [120H] integral intensity of PAMAM G3 resonance centered at 2.20 ppm (Figure 1B).

Analogously, the positions and intensities of the S resonances in ternary conjugate G3^2B2C4S12gl^ enabled the determination of the stoichiometry of the conjugate, containing average 4S substituents. In this ^1^H NMR spectral quantitation, another ^1^H internal reference, PAMAM resonance, was used, the one centered at 2.60, because the resonance at 2.20 ppm was overlapped with celecoxib-derived methyl 12c resonance (Figure 1C). The ^1^H NMR spectrum of G3^2B2C4S12gl^ conjugate showed very broad residual HDO resonance, which precluded a detailed spectrum analysis within 3.5–4.5 ppm region.

Celecoxib (C) resonances in the ^1^H NMR spectrum in DMSO-d_6_ (Figure 1E) were identified on the basis of C resonances integral intensity and COSY spectrum (not shown). All the aromatic proton resonances, except 15c and 17c of C in the ^1^H NMR spectrum of both G3^2B6C8gl^ and G3^2B2C4S12gl^, were grouped within 7.3–7.4 ppm. The integration of these resonances originated from seven protons of C enabled to determine the number of C substituents equal to six and two in G3^2B6C8gl^ and G3^2B2C4S12gl^, respectively (Figure 1D,C, respectively).

In conclusion, we obtained the binary conjugates G3^2B4S14gl^ and G3^2B6C8gl^, as well as the ternary G3^2B2C4S12gl^ with remaining amine groups mono- or di-substituted with *R*-glycidol or unsubstituted at around 12, 16, and 12, respectively (Scheme 1). Glycidylation of conjugates was performed in order to increase a solubility of the conjugates in water but also to eradicate free amine groups, which are responsible for a systemic toxicity of PAMAM dendrimers, which increases considerably with PAMAM generation and amount of amine groups [30].

Although non-glycidylated analogues are well soluble in DMSO, they precipitate upon dilution with water. Surprisingly, the glycidylated conjugates obtained eventually were found to be better soluble in water and kept their molecular size at the molecular level, as determined by DLS method for 0.1 mM solution (Table 1).

We chose *R*-glycidol to derivatize the binary and ternary conjugates because we noticed more effective internalization of G3 and G2 PAMAM dendrimers covered with *R*-glycidol in comparison with those covered with *S*-glycidol into keratinocyte (HaCaT) and especially squamous carcinoma (SCC-15) cells in vitro [21].

The molecular size of conjugates, d(N), depends on primary amine groups available for protonation, which is achieved already at pH 5. The molecular size of the carrier, G3^2B14gl^, was 3.3 nm (Table 1, Figure S5). The binary G3^2B6C8gl^ and G3^2B4S14gl^ sizes were 4.0 and 6.5, respectively, while a diameter of ternary G3^2B2C4S12gl^ molecule was 6.9 nm at pH 5 measured as d(N) at 0.05 mM concentration. In all cases, the volume-averaged diameter, d(V), was slightly larger than d(N), which is a regular feature of the conjugates. However, the ternary G3^2B2C4S12gl^ showed substantial association at pH 7, with d(N) 97 nm and d(V) around 150 nm as measured for 0.1 mM solution. The obtained results were important for further biological tests; the solutions used there were 10 times and more diluted. Thus, we assumed that all the conjugates were dispersed into the monomolecular level and the concentrations of all the conjugates were actual. In the case of G3^12S^, the molecules remained associated both in pH 7 and pH 5 with particles sized at least 25 nm (Figure S5, Table 1).

Consistently, the zeta potential values for all conjugates, except G3^12S^, fell into 8–38 mV, which are the numbers suggesting that high affinity of the conjugate molecules into negatively charged cell membranes is achievable (Figure S6, Table 1).

### 3.2. Conjugates Cytotoxicity

Dendrimer G3^2B14gl^ revealed rather low cytotoxicity for both cancer lines as estimated with neutral red (NR) assay: IC_50_ in order of 200 µM and 70 µM in SCC-15 and U-118 MG, respectively, and much higher cytotoxicity in BJ cells with IC_50_ fourfold lower in both assays (Table 2, Figure S7). This may indicate that under applied conditions, the G3^2B14gl^ penetrated the cell membrane without significant damage, whereas it entered the mitochondria and disrupted their transmembrane potential since the XTT assay indicated significantly lower IC_50_ values (Table 2, Figure S7).

**Figure 2:**
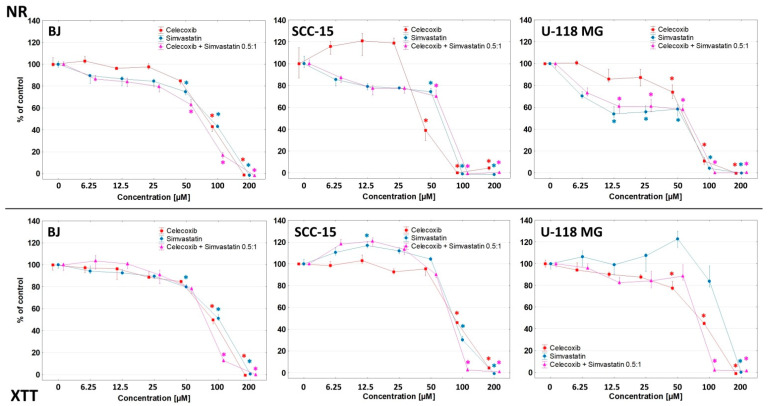
Effect of single drugs celecoxib, simvastatin, and 0.5:1 mixture of celecoxib and simvastatin on normal BJ and cancer SCC-15 and U-118 MG cell viability, estimated with NR and XTT assays after 24 h incubation. Cell viability is presented as medians of a percent against non-treated control (control expressed as 100%). The whiskers are lower (25%) and upper (75%) quartile ranges. * *p* ≤ 0.05; Kruskal–Wallis test (against non-treated control).

The cytotoxicity of investigated repurposed drugs alone evaluated in all investigated cell lines revealed that their activities estimated with XTT assay as being in the order of 100 µM for both drugs and were higher than data published by the others for different cancer cell lines (Table 2). Gehrke et al. estimated the IC_50_ for C and S in the head and neck squamous carcinoma cell lines PE/CA-PJ 41 and HLaC78 using the MTT assay as 40 µM and 50 µM, respectively [19]. Comparable with those results are our data obtained with NR assay for C in both investigated cancer lines (IC_50_ = 48 µM for SCC-15) and (IC_50_ = 60 µM for U-118 MG) and with S (IC_50_ = 57 µM) in SCC-15 cells. Interestingly, the higher cytotoxicity of S (IC_50_ = 26 µM) was observed in glioma U-118MG cells. This may be explained by the observation including the breast (MCF-7 and SKBr-3), prostate (LNCaP and PC-3), colon (Caco-2 and HCT-116), skin (SCC-M7 and SCC-P9), and the lung cancer cell lines (Calu-3 and Calu6) that S inhibited the cell growth more efficiently in less differentiated tumors that spread faster, as in case of glioblastoma. The observed cytotoxicity for investigated cells was measured after treatment with 1 to 20 µM S for a prolonged period of 72 h [32]. The combination of treatment with both drugs by Gehrke et al. decreased tumor cell viability significantly more as compared to C and S alone, wherein a small additive effect and no synergistic effect were observed [19]. Our data revealed a higher additive effect of both drugs with XTT assay, but almost no effect with NR assay (Table 2). This may be explained by the data of Fujiwara et al., who reported that statins promote cell death by increasing the activation of caspases-3/-9, inducing pro-apoptotic protein (Bim) expression, arresting the cell cycle at the G1 phase, and decreasing the mitochondrial membrane potential (Δψm) [33]. Results obtained with either drug alone or both provided two interesting observations. The first is the higher resistance of fibroblasts to C and S as estimated by both assays as compared to cancer lines. The second is the highest cytotoxicity of S alone and in the combination with C in U-118 MG cells (IC_50_ = 26 µM and 38 µM, respectively; Figure 2).

Menter et al., 2017 [32] observed a time-dependent two-phased response in tumor cells to simvastatin. The first phase within the 6 h to 24 h involved changes in cell morphology, whereas the second phase between 24 and 72 h involved the loss of plasma membrane integrity as an effect of the cholesterol depletion. This is connected with disruption of the lipid rafts and expression of the caveolin-1 responsible for cellular transport and signaling, leading to the induction of apoptosis [32]. Thus, as was mentioned earlier, the NR assay showed higher sensitivity of U-118 MG to S for disintegration of the cellular membrane than SCC-15 and BJ cells.

**Figure 3:**
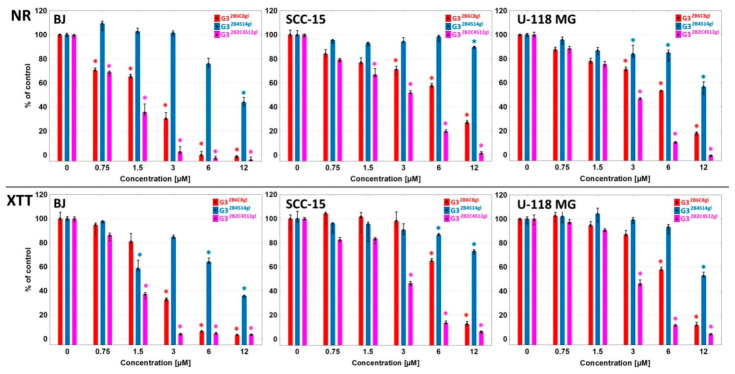
Cytotoxicity of single conjugates G3^2B6C8gl^, G3^2B4S14gl^, and combined conjugate G3^2B2C4S12gl^ for normal (BJ) and cancer (SCC-15) and (U-118 MG) cells after 24 h treatment, estimated with neutral red (NR) or XTT assay. Cell viability is presented as medians of a percent against non-treated control (control expressed as 100%). The whiskers are lower (25%) and upper (75%) quartile ranges. * *p* ≤ 0.05; Kruskal–Wallis test (against non-treated control).

Conjugation of the G3^2B14gl^ with either drug alone or both dramatically increased their cytotoxicity as compared with drugs alone with much lower differences depending on used assays and investigated cancer cell lines (Figure 3). Estimated IC_50_ were in the order of 6–12 µM with lower values for G3^2B6C8gl^ in BJ cells (about 2 µM). This result was 10- to 50-fold lower as compared to both drugs alone (Table 2). A comparison of the IC_50_ values of the conjugates and the equivalent of the drugs (calculated concentration of introduced drugs) showed that the toxicity of the conjugates was not as impressively higher than that of the drugs themselves, but still the conjugates showed about 2-8-fold higher activity than the drug equivalents (Table 2). Conjugate with both drugs G3^2B2C4S12gl^ further increased the cytotoxicity of constructs as compared with conjugates of a single drug by 2–8-fold, with IC_50_ in the order of 1 µM for BJ and 3 µM for both cancer cell lines. This amounted to a 35–50-fold increase of cytotoxicity as compared to both drugs alone (Table 2). The negative finding is the lack of selectivity of obtained conjugate that is more toxic (threefold) for normal fibroblasts than for both cancer lines. However, this may have meaning for cancer-associated fibroblasts (CAF) constituting the tumor extracellular matrix (TEM) now recognized as a substantial factor for cancer progression and considered as a potential therapeutic and diagnostic target [34]. The interaction between tumor cells and the TEM plays a crucial role in tumor initiation, progression, metastasis, and response to therapy. The S has been shown to induce metabolic reprogramming in TEM and to target the immune microenvironment through cytokines or chemokines [35].

## 4. Conclusions

The presented results documented the very high effectiveness of obtained biotinylated and *R*-glycidylated PAMAM dendrimer conjugated with both C and S (G3^2B2C4S12g^) as dramatically decreasing the squamous carcinoma SCC-15 and glioblastoma U-118 MG cell viability at very low concentrations with IC_50_ equal to about 3 µM. This makes it a prospective targeted drug carrier in malignant cancer, including glioblastoma therapy with decreased side effects of both drugs observed at concentrations applied in the clinics.

## Figures and Tables

**Table 1:** Size of conjugates averaged by volume (d(V)) and by number of molecules (d(N)) and zeta potential values ± standard deviation determined by DLS analysis.

Compound	Size (nm)				Zeta Potential (mV)	
	pH 7		pH 5		pH 7	pH 5
	d(V)	d(N)	d(V)	d(N)		
G3^2B14gl^	2.1 ± 0.49	1.8 ± 0.42	5.0 ± 0.96	3.3 ± 0.85	9.6 ± 0.89	7.8 ± 2.51
G3^2B6C8gl^	7.8 ± 3.56	5.7 ± 2.35	5.8 ± 1.59	4.0 ± 1.46	18.6 ± 3.70	34.6 ± 2.52
G3^2B4S14gl^	10.3 ± 1.67	8.6 ± 1.44	9.5 ± 1.97	6.5 ± 1.92	37.7 ± 3.82	36.5 ± 4.11
G3^2B2Cel4S12gl^	155 ± 5.77	86.7 ± 6.83	8.5 ± 2.19	6.9 ± 2.14	30.3 ± 0.73	32.5 ± 3.14
G3^12S^	85.8 ± 1.83	57.2 ± 2.96	44.2 ± 1.99	25.3 ± 7.07	42.4 ± 1.77	32.9 ± 2.84

**Table 2:** Cytotoxicity of investigated repurposed drugs alone and their conjugates with G3^2B14gl^ estimated for the normal BJ and cancer SCC-15 and U-118 MG cells, expressed as half maximal inhibitory concentration (IC_50_) in micromolar concentration (medians). Data were estimated with NR and XTT assay after 24 h incubation and calculated with AAT Bioquest IC_50_ calculator [31]. *a*—concentration of introduced C, *b*—concentration of introduced S.

Assay	Cell Line	Conjugate/Carrier Alone/Drug Alone (IC_50_ µM)						
		G3^2B6C8gl^	G3^2B4S14gl^	G3^2B2C4S12gl^	G3^2B14gl^	C	S	C + S 0.5:1 Ratio
NR	BJ	1.90	9.99	1.10	53.60	88.68	90.14	63.40
		11.40 *^a^*	39.96 *^b^*	2.20 *^a^*				
				4.40 *^b^*				
	SCC-15	6.65	>12.00	6.69	>200.00	47.48	56.54	56.45
		39.9 *^a^*	>48.00 *^b^*	13.38 *^a^*				
				26.76 *^b^*				
	U-118 MG	5.86	16.90	2.73	>200.00	65.57	26.19	37.65
		35.16 *^a^*	67.60 *^b^*	5.46 *^a^*				
				10.92 *^b^*				
XTT	BJ	2.40	8.75	1.28	23.45	100.13	100.86	66.00
		14.40 *^a^*	35.00 *^b^*	2.56 *^a^*				
				5.12 *^b^*				
	SCC-15	6.98	>12.00	2.98	64.58	97.13	84.38	60.14
		41.88 *^a^*	>48.00 *^b^*	5.96 *^a^*				
				11.92 *^b^*				
	U-118 MG	6.49	12.27	2.91	62.65	93.74	112.33	70.67
		38.94 *^a^*	49.08 *^b^*	5.82 *^a^*				
				11.64 *^b^*				

## Data Availability

Data supporting reported results can be provided by authors upon request.

## Supplementary Materials

The following supporting information can be downloaded at https://www.mdpi.com/article/10.3390/cancers14030714/s1, Figure S1: Progress of reaction between S and en monitored by the ^1^H NMR spectroscopy. Figure S2: COSY spectrum of G3^12S^ in DMSO-d_6_. Figure S3: COSY spectrum of simvastatin in DMSO-d_6_. Figure S4: COSY spectrum of G3^2B4S12gl^ in DMSO-d_6_. Figure S5: Size of conjugates by DLS. Figure S6: Zeta potential of conjugates. Figure S7: Cytotoxicity of G3^2B14gl^ carrier for BJ, SCC-15, and U-118 MG cells. Table S1: The ^1^H and ^13^C NMR chemical shifts of simvastatin (S); celecoxib (C); and G3^12S^, G3^2B4S14gl^, G3^2B2C4S12gl^, and G3^2B6C8gl^ conjugates in DMSO-d_6_.
