# Supplementary material for: Synthesis of Biotinylated PAMAM G3 Dendrimers Substituted with R-Glycidol and Celecoxib/Simvastatin as Repurposed Drugs and Evaluation of Their Increased Additive Cytotoxicity for Cancer Cell Lines
Source: Cancers (Basel). 2022 Jan 29;14(3):714. doi: 10.3390/cancers14030714 (PMC8833738; doi:10.3390/cancers14030714)
Supplement: Supplementary file 1 [file cancers-14-00714-s001.zip › cancers-1547731-supplementary.pdf]

SUPPLEMENTARY MATERIALS

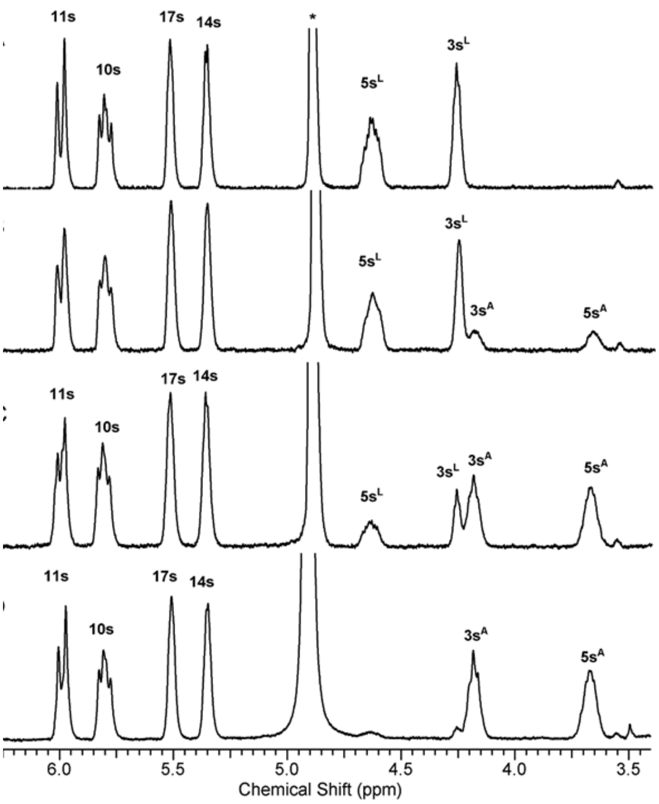

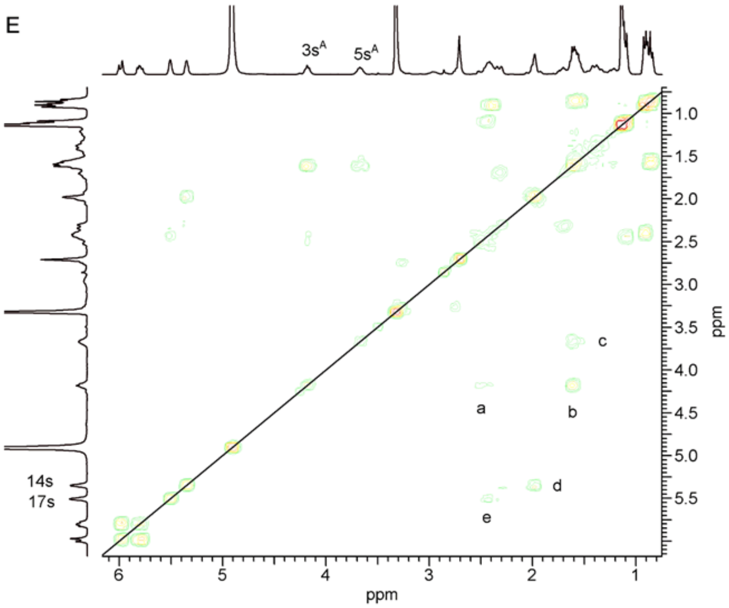

Figure S1. Progress of reaction between S and en monitored by the ^{1}H NMR spectroscopy. The spectra were recorded in CD_3OD after: mixing of reagents (A); 1 hour reaction at RT (B); 2 hours refluxing (C); 4 hours refluxing (D); ^{1}H- ^{1}H- COSY spectrum of the solution after 4 hours reflux (E).
Labeled cross-peaks correspond to three bonds scalar coupling: a – 3H/2H; b – 3H/4H; c – 5H/6H; d – 14H/13H; e – 17H/16H

Figure S1. Progress of reaction between S and en monitored by the 1H NMR spectroscopy

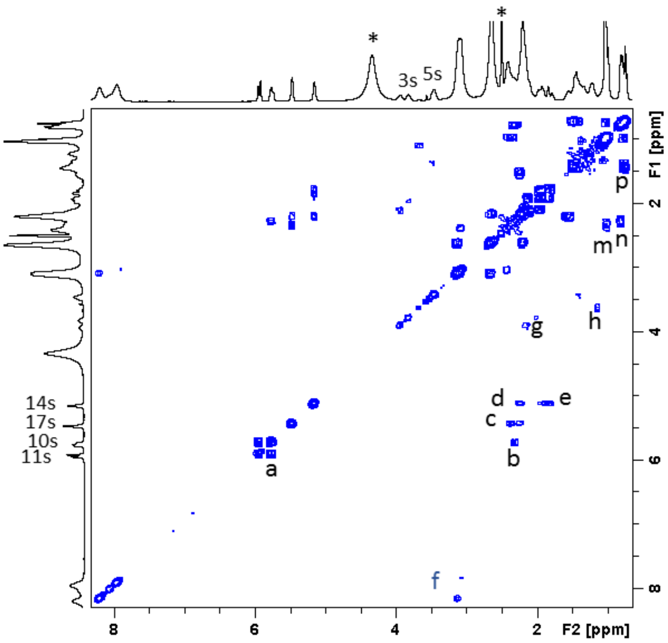

Figure S2. COSY spectrum of G3[12S] in dmso-d_6.
The relevant cross-peaks are:
a – 10s/11s
b – 10s/9s
c – 17s/16s
d – 14s/13s
e – 14s/15s
f – N<u>H</u>/C<u>H</u>$_2$ (PAMAM)
g – 3s/2s
h – 5s/4s
m - 24s/16s
n – 25s/9s
p – 21s/20s
The resonances of protons 2(2’), 4(4’), 6(6’), 7(7’), 8, 9, 13, 15(15’), and 16 are partially overlapped within the 3.57-1.14 ppm region. <u>H</u>DO and residual dmso-d_6 proton quintet are labelled with asterisks

Figure S2. COSY spectrum of G312S in DMSO-d6

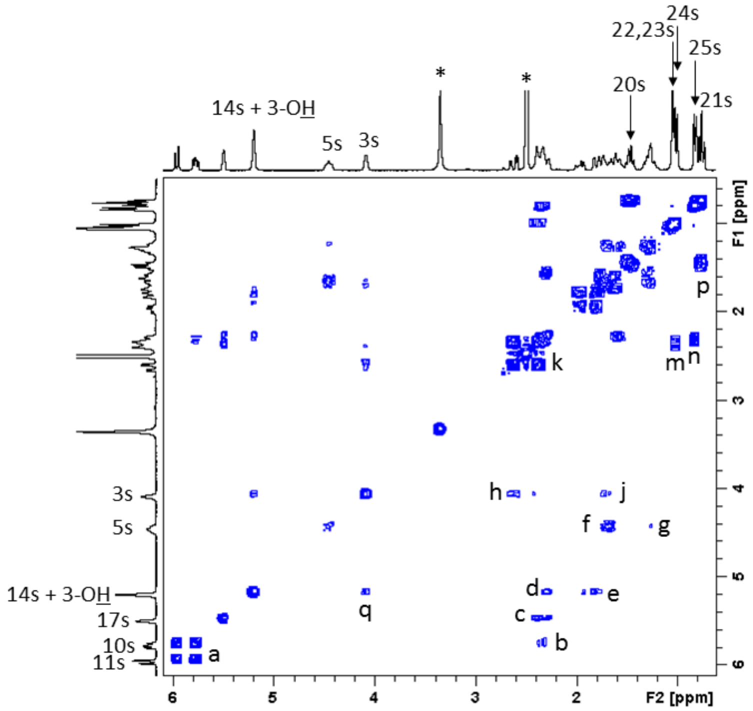

Figure S3. COSY spectrum of simvastatin in dmso.
The relevant cross-peaks are:
a – 10s/11s
b – 10s/9s
c – 17s/16s
d – 14s/13s
e – 14s/15s
f – 5s/4s
g – 5s/6s
h – 3s/2s
j – 3s/4s
k – 2s/2's
m - 24s/16s
n – 25s/9s
p – 21s/20s
q – 3s/3-OH
The resonances of protons 2(2'), 4(4'), 6(6'), 7(7'), 8, 9, 13, and 15(15') are overlapped within the 2.7-1.2 ppm region. HDO and residual dmso-d_6 proton quintet are labelled with asterisks

Figure S3. COSY spectrum of simvastatin in DMSO-d6.

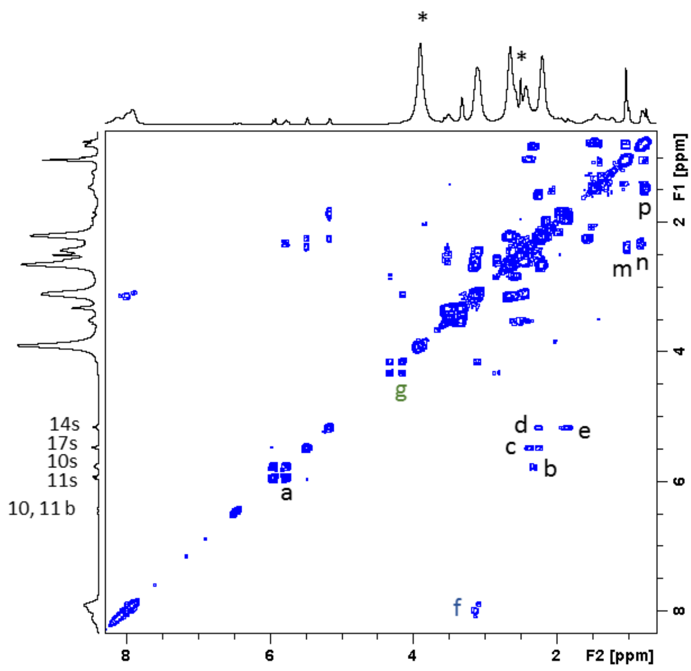

COSY spectrum of G3^{2B4S14gl} in dmso-d_6.
The relevant cross-peaks are:
a – 10s/11s
b – 10s/9s
c – 17s/16s
d – 14s/13s
e – 14s/15s
f – NH/CH_2 (PAMAM)
g – 8b/9b (biotin residue)
m - 24s/16s
n – 25s/9s
p – 21s/20s
The resonances of protons 2(2'), 3, 4(4'), 5, 6(6'), 7(7'), 8, 9, 13, 15(15'), and 16 are overlapped within the 3.57-1.14 ppm region. HDO and residual dmso-d_6 proton quintet are labelled with asterisks

Figure S4. COSY spectrum of G32B4S12gl in DMSO-d6.

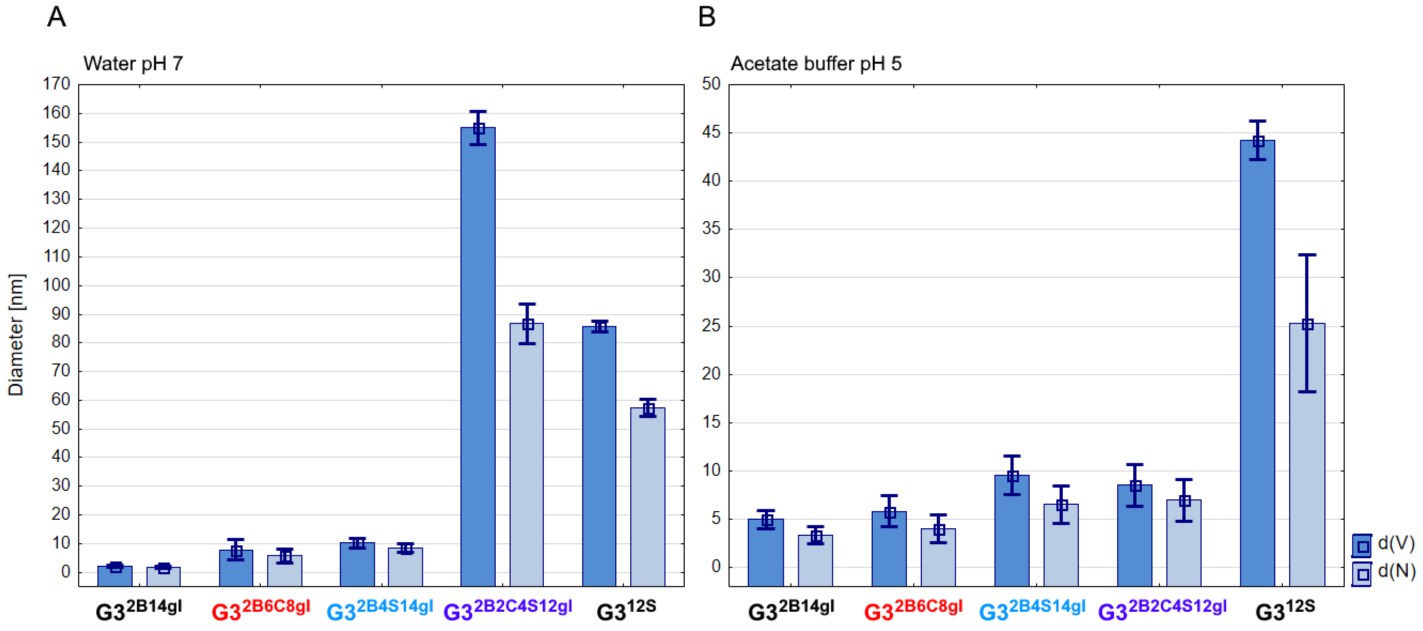

Figure S5. Size of conjugates by DLS.

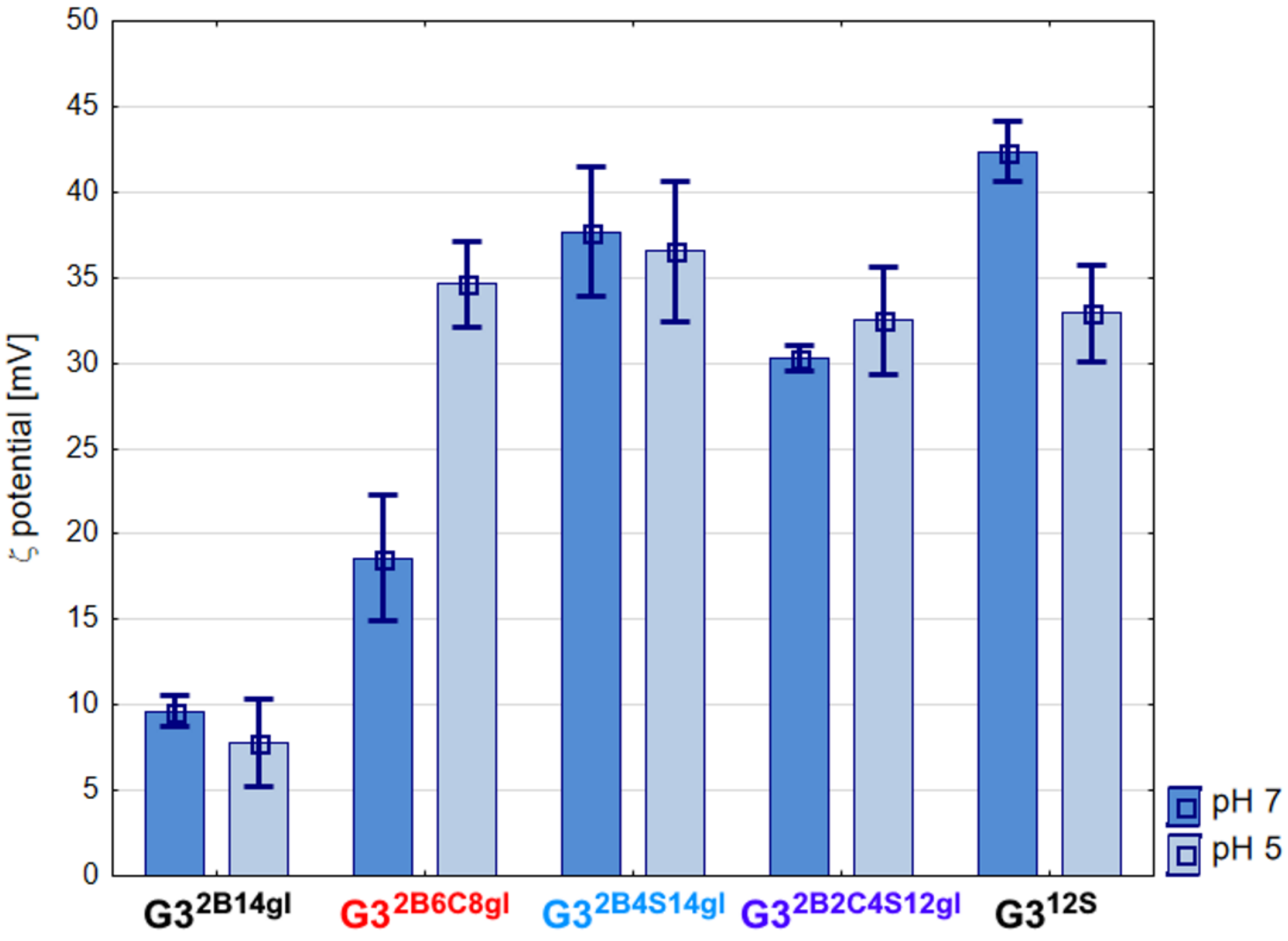

Figure S6. Zeta potential of conjugates.

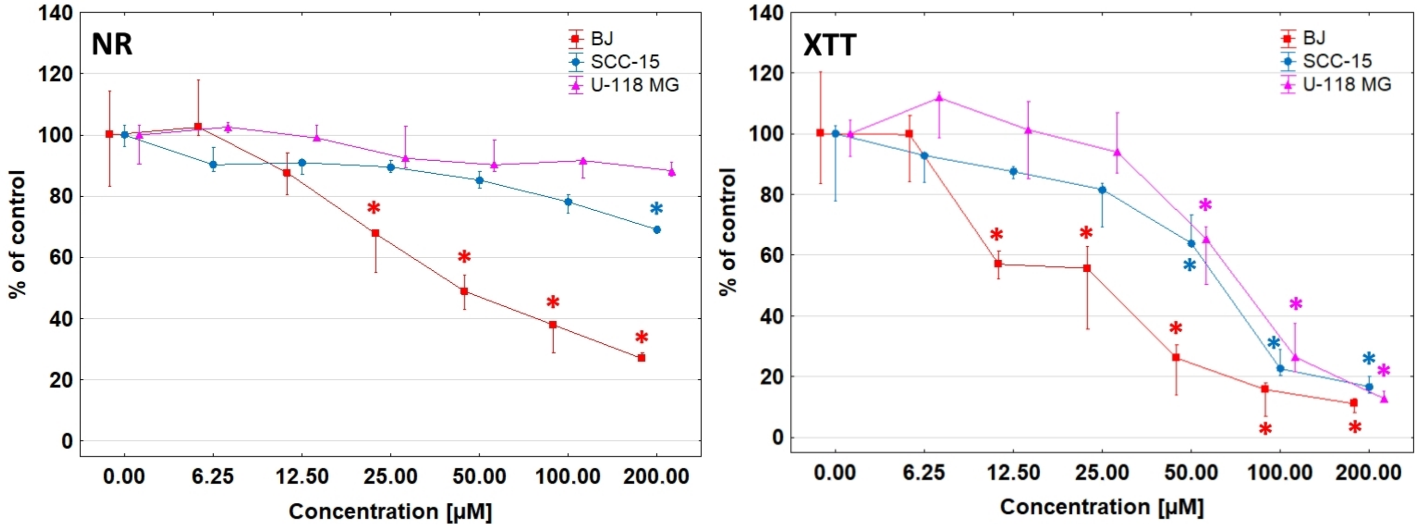

Figure S7. Cytotoxicity of G32B14gl carrier for BJ, SCC-15, and U-118 MG cells, Table S1: The 1H and 13C NMR chemical shifts of simvastatin (S), celecoxib (C), and G312S, G32B4S14gl, G32B2C4S12gl, and G32B6C8gl conjugates in DMSO-d6

Table S1

The ^{1}H and ^{13}C NMR chemical shifts of simvastatin (S), celecoxib (C), and $G3^{12S}$, $G3^{2B4S14gl}$, $G3^{2B2C4S12gl}$, and $G3^{2B6C8gl}$ conjugates in DMSO-d_6. The annotation nd means not detected due to limited concentration of the species or not assigned due to overlap with strong resonances from PAMAM carrier, which are not listed here (for spectral assignment of PAMAM and biotin resonances see [17].

Species→	S		$G3^{12S}$		$G3^{2B4S14gl}$		$G3^{2B2C4S412gl}$		$G3^{2B6C8gl}$		C	
Locant ↓	^{13}C	^{1}H	^{13}C	^{1}H	^{13}C	^{1}H	^{13}C	^{1}H	^{13}C	^{1}H	^{13}C	^{1}H
1s	170.65	-	172.03	-	172.3	-	171.96	-				
			171.54									
2s	38.95	2.62	44.34	1.35	44.34,	nd	45.01	nd				
		2.37	44.51		44.51							
3s	61.69	4.10	66.78	3.93	69.31	nd	69.33	nd				
			67.15	3.83								
4s	36.63	1.60	45.10	2.12	45.10,	nd	45.01	nd				
		1.54	44.97	1.97	44.97							
5s	76.30	4.46	69.34	3.46	68.20	nd	66.82	nd				
6s	32.84	1.26	34.82	1.37	34.82	nd	34.87	nd				
		1.71										
7s	24.12	1.28	24.64	nd	24.64	nd	24.66	nd				
8s	35.72	1.65	38.74	nd	36.98	nd	36.55	nd				
9s	30.59	2.33	30.72	nd	30.69	nd	30.69	nd				
10s	133.45	5.77	133.72	5.76	133.75	5.75	133.75	5.75				
11s	128.69	5.96	128.67	5.93	128.60	5.94	128.60	5.94				
12s	131.93	-	132.16	-	132.18	-	132.18	-				
13s	36.97	2.29	36.96	nd	36.96	nd	36.97	nd				
			37.04		37.04							
14s	68.03	5.20	68.12	5.15	68.17	5.16	68.13	5.15				
15s	32.47	1.84	32.47	nd	32.47	nd	32.38	nd				
		1.92										
16s	27.15	2.39	27.15	nd	27.20	nd	27.20	nd				
17s	129.61	5.50	129.36	5.47	129.40	5.47	129.35	5.46				
18s	177.05	-	177.05	-	177.03	-	177.02	-				
19s	42.85	-	42.80	-	42.80	-	42.77	-				
20s	32.93	1.47	32.99	nd	32.97	nd	33.03	nd				
21s	9.60	0.76	9.50	0.75	9.55	0.77	9.57	0.75				
22s	24.88	1.05	24.81	1.03	24.78	1.03	24.75	1.02				
23s	24.93	1.05	24.82	1.03	24.91	1.03	24.84	1.02				
24s	23.22	1.01	23.12	1.00	23.22	1.03	23.20	1.09				
25s	14.07	0.82	14.05	0.80	14.04	0.79	14.66	0.79				
1g					52.86	nd	55.81	nd	55.92	nd		
2g					70.72	3.50	70.79	3.48	61.61	3.40		
3g					64.98	3.31	64.99	3.31	59.62	3.25		
3c							nd	nd	nd	-	142.62(q)	-
4c							nd	7.16	111.39	7.15	106.66	7.20
5c							125.20	-	125.94	-	125.92	-
6c							145.45	-	147.13	-	145.81	-
7,11c							129.10	7.16	129.06	7.15	129.27	7.20
8,10c							129.80	7.16	129.80	7.15	129.89	7.20
9c							139.39	-	139.35	-	139.65	-
12c							21.21	2.12	21.24	2.27	21.31	2.32
13c							145.53	-	145.46	-	144.50	-
14,18c							125.90	7.31	125.26	7.30	126.47	7.54
15,17c							128.15	7.75	128.13	7.73	127.30	7.87
16c							140.18	-	141.95	-	141.58	-
20c							163.28	-	163.28	-		
21c							nd	nd	nd	nd		
22c							nd	nd	nd	nd		
23c							172.83	-	172.74	-		
